# Transformation and causes of death in follicular lymphoma: A Finnish nationwide population‐based study

**DOI:** 10.1111/bjh.70181

**Published:** 2025-09-25

**Authors:** Ilja Kalashnikov, Taina Reunamo, Tomas Tanskanen, Leevi Viisanen, Nea Malila, Sirkku Jyrkkiö, Sirpa Leppä

**Affiliations:** ^1^ Research Programs Unit, Faculty of Medicine University of Helsinki Helsinki Finland; ^2^ Department of Oncology Helsinki University Hospital Comprehensive Cancer Centre Helsinki Finland; ^3^ Wellbeing Services County of Southwest Finland, Department of Oncology Turku University Hospital and University of Turku Turku Finland; ^4^ Finnish Cancer Registry Cancer Society of Finland Helsinki Finland

**Keywords:** excess mortality, follicular lymphoma; real word data, relative survival, transformation

## Abstract

Follicular lymphoma (FL) is a common, indolent lymphoma, and patients with FL typically have a good prognosis. However, they may experience histological transformation into aggressive large B‐cell lymphoma. We conducted a nationwide population‐based study to estimate the risk of transformation in FL, considering different FL grades, and studied the relative survival (RS) of patients diagnosed with FL in Finland from 1995 to 2018. We identified a total of 4014 patients with newly diagnosed grade 1–3A FL. The median age at diagnosis was 64 years, and 55% of patients were female. The cumulative incidence of transformation across the entire cohort was 8.4% at 10 years (95% confidence interval [CI], 7.5–9.5). The 10‐year RS was 78% for the whole cohort and showed improvement over time. Transformation was associated with a significantly increased risk of death (hazard ratio [HR] 5.0; 95% CI, 4.2–6.0; *p* < 0.001). Grade 3A was associated with higher excess mortality compared to patients with low‐grade FL. Lymphoma was the most common cause of death. We conclude that, although the 10‐year RS was relatively good, grade 3A FL and transformation led to significantly higher mortality compared to low‐grade FL or no transformation. Our results also indicate a reduction in excess mortality over time.

## Graphical abstract

This large, nationwide population‐based study showed a 10‐year relative survival (RS) of follicular lymphoma (FL) at 78%. Prognosis improved over time. The 5‐ and 10‐year cumulative transformation incidences were 4.6% and 8.4% respectively. Transformation, grade 3A FL and age were associated with excess mortality. Lymphoma was the most common cause of death.

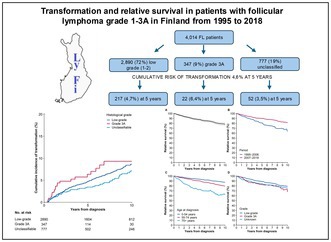

## INTRODUCTION

Follicular lymphoma (FL) is the second most common lymphoma and the most common indolent lymphoma.^1^, ^2^ At the beginning of the 21st century, the introduction of rituximab, an anti‐CD20 monoclonal antibody, to the treatment of FL improved remission rates. Thus, the prognosis of FL has improved over the decades,^2^ with 10‐year overall survival (OS) currently approximately 70%–80%.^1^, ^2^ However, FL remains incurable, with relapses occurring even decades later.

FL arises from B cells and is graded from 1 to 3 based on the average number of centroblasts per high‐power microscopy field. Grade 3B FL, marked by centrocytes, is considered aggressive lymphoma. In contrast, treatments for indolent grades 1–3A FL vary from surveillance and local radiotherapy to single‐agent rituximab and immunochemotherapy, depending on tumour burden and the patient's symptoms.

There is no consensus on whether grade 3A FL patients have a worse prognosis than those with grades 1–2 and require more aggressive treatment. Some studies suggest that grade 3A FL patients benefit from anthracycline‐based chemotherapy like those with grade 3B disease,^3^, ^4^, ^5^ while other studies indicate that the difference is negligible.^6^, ^7^ Thus, in the latest update of the 5th edition of the World Health Organization (WHO) Classification of haematolymphoid tumours, grading of classic FL is no longer mandatory,^8^ partly due to the poor reproducibility of grading.^9^ In contrast, FL grading is still included in the International Consensus Classification of Mature Lymphoid Neoplasms.^10^

FL can transform into aggressive B‐cell lymphoma, typically diffuse large B‐cell lymphoma (DLBCL) or high‐grade B‐cell lymphoma (HGBCL), with a reported overall transformation rate of 10%–37% and an annual incidence of 2%–3%.^11^, ^12^, ^13^

We conducted a nationwide, population‐based retrospective study to evaluate transformation risk in FL, including an analysis by histological grade. Additionally, we evaluated the relative survival (RS) of patients diagnosed with FL in Finland between 1995 and 2018 and compared excess mortality between patients with transformation and those without.

## METHODS

### Data sources

The data were obtained from the Finnish Cancer Registry (FCR), which serves as a statistical and epidemiological research institute. According to special legislation, physicians, hospitals and pathology and haematology laboratories are required to report new cancer cases to the FCR without patient consent; therefore, the FCR has maintained nationwide data on all cancer diagnoses in Finland since 1953. The reports include a unique personal identity code enabling reliable follow‐up for patients and linkage between national registries. Information on the deaths of cancer patients is provided annually by Statistics Finland and linked to the FCR data using this personal identity code.

Histologically verified transformations are also reported to the FCR as obligatory by the pathological departments and are recorded as new entities in the FCR, and can therefore be identified in patients with a history of haematological malignancy.

Since 2007, cancer coding has followed the International Classification of Diseases for Oncology, 3rd Edition (ICD‐O‐3), consistent with the WHO Classification of Tumours of Haematopoietic and Lymphoid Tissues, 4th Edition.^14^ In 2007, previous codes from the Manual of Tumor Nomenclature and Coding were converted to ICD‐O‐3.^15^, ^16^ The FCR has high overall coverage; estimated coverage for lymphoid cancers is 94%, and 99% of cases are morphologically verified.^17^

### Patients

From the FCR database, patients diagnosed with FL grade 1–3 between 1 January 1995 and 21 December 2018 were retrieved using ICD‐O‐3 morphology codes 9690/3, 9691/3, 9695/3 and 9698/3. Patients with the ICD‐O‐3 code 9591/3, indicating lymphoma not otherwise specified (NOS), were reviewed. The diagnosis and transformations to aggressive lymphoma were confirmed by manually reviewing free‐text sections of the linked pathology and clinical reports. No histopathological review was performed. The cohort was stratified based on histological grade from the initial tissue biopsy into low‐grade (grades 1–2), grade 3A and unclassified FL for cases where the grade was undetermined from the registry data.

Patients with grade 3B FL, composite lymphoma, diagnoses confirmed only at autopsy or post‐mortem, and cases with unclear diagnoses in the registry data were excluded. Thus, 4014 patients with newly diagnosed grade 1–3A FL in Finland from 1995 to 2018 were included.

Incident transformation was defined as morphologically verified large B‐cell lymphoma or HGBCL diagnosed at least 3 months after the initial FL diagnosis.

Clinical data, including sex, age at diagnosis, date of last follow‐up, vital status at the end of follow‐up (31 December 2018) and date of transformation, were collected from the registry. The statistical underlying cause of death for deceased FL patients was retrieved from Statistics Finland. Causes of death were determined according to the International Classification of Diseases by the WHO 10th revision^18^ and grouped into three categories: any lymphoma, secondary malignant neoplasm (SMN) or other cause. No patients were lost to follow‐up. The general population mortality rates for Finland were obtained from Statistics Finland.

This study was conducted in accordance with the Declaration of Helsinki. The study was approved by the National Institute for Health and Welfare (Dnro THL/1441/5.05.00/2019), Statistics Finland (Dnro TK‐53‐1172‐19) and the Helsinki University Hospital Institutional Review Board. Written informed consent was waived due to the retrospective nature of the study and the de‐identification of the patient information. All methods were carried out following the WHO and ICD‐O‐3 guidelines and recommendations.

### Statistical analysis

The start of follow‐up was the date of the FL diagnosis. OS was estimated using the Kaplan–Meier method, and hazard ratios (HRs) for total mortality and incident transformation were estimated using multivariable Cox regression models with a 95% confidence interval (CI). The Aalen–Johansen estimator was used to calculate the cumulative incidence of transformation, treating death from other causes as a competing event, as well as the cumulative incidence for the competing risks of cause of death.

RS was estimated using the Ederer II method with internal age standardization (age groups: 0–44, 45–54, 55–64, 65–74 and ≥75 years at diagnosis).^19^ Follow‐up time was divided into monthly intervals. Age‐specific RS was estimated for three age groups based on the age at diagnosis (0–54, 55–74 and ≥75 years). The complete analysis included all person‐time and deaths from 1995 to 2018, while period analyses were conducted separately for 1995−2006 and 2007−2018, using left‐truncated data for the later period.^20^

To estimate HRs for excess mortality, we used multivariable flexible parametric survival models.^21^ Expected mortality was derived from the general population mortality rates of Finland, categorized by 1‐year age group, calendar year and sex. The baseline excess hazard rate was modelled with four degrees of freedom.^22^ To address immortal time bias, the transformation was modelled as a time‐varying covariate that could change during follow‐up. To accommodate non‐proportional hazards, we fitted separate models that also factored in time from transformation. After dividing the follow‐up period into monthly intervals, the time‐dependent effect of transformation was modelled using a restricted cubic spline with two degrees of freedom.

The Wilcoxon rank‐sum test and Kruskal–Wallis test were used to compare the age at diagnosis among patient groups.

Statistical analyses were performed using R version 4.0.2 (R Foundation for Statistical Computing, Vienna, Austria), along with the packages survival 3.1–12, rstpm2 1.5.2, Epi 2.44 and popEpi 0.4.8.

## RESULTS

### Patients

We identified 4014 patients with newly diagnosed FL in Finland between January 1995 and December 2018. The median age at diagnosis was 64 years (interquartile range [IQR], 55–72; range, 14–100). There was a slight female predominance (55%), with females being older than males at diagnosis (median age, 64 vs. 62; *p* < 0.001). Patients diagnosed between the years 2007 and 2018 were older at diagnosis than those diagnosed between the years 1995 and 2006 (65.7 years vs. 59.2 years) (Table S1).

Of the cohort, 72% (*n* = 2890) had low‐grade FL, 9% (*n* = 347) had grade 3A and 19% (*n* = 777) were unclassified. Among patients diagnosed in 2007–2018, 12% had grade 3A FL compared to only 2.0% among patients diagnosed in 1995–2006 (Table S1). Grade 3A FL patients were slightly older at diagnosis than those with low‐grade or unclassified FL patients (66.0 vs. 64.0 vs. 64.0; *p* < 0.001). Median follow‐up was 8.9 years (IQR, 4.0–14.5; range 0–24 years).

### Risk of transformation

**FIGURE 1:**
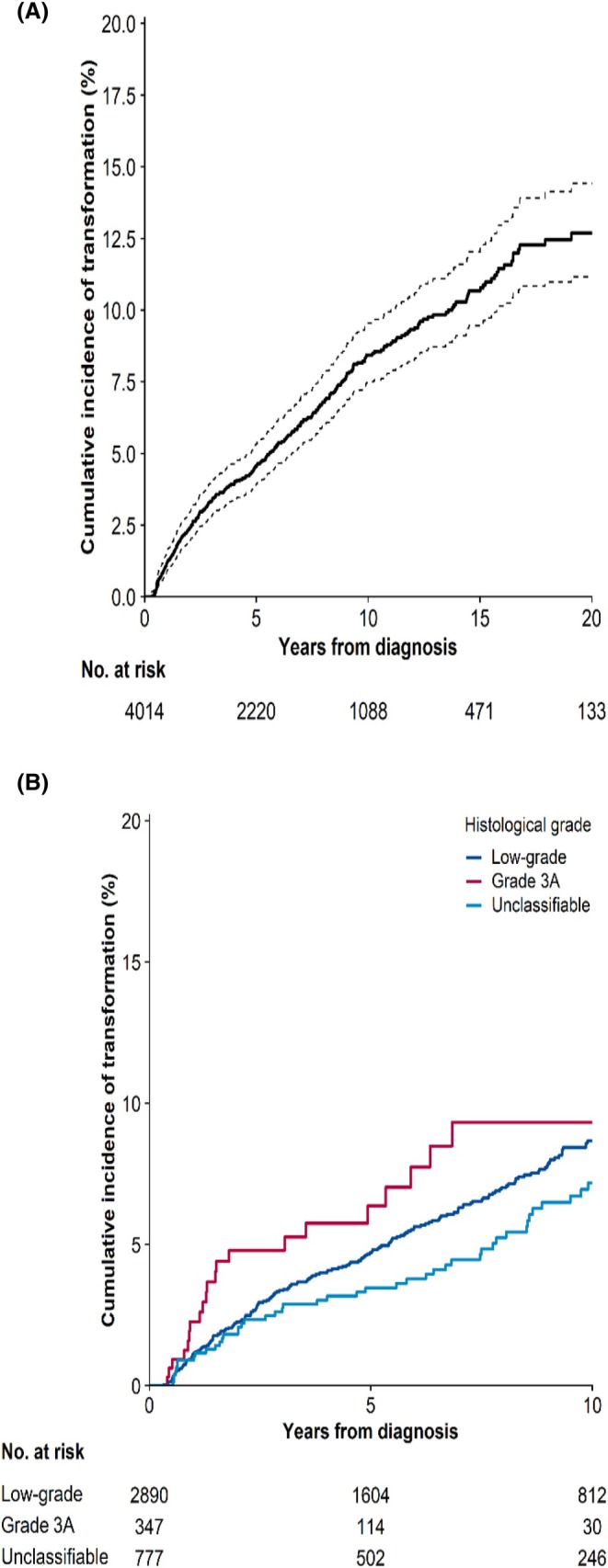
Cumulative incidence of transformation in patients diagnosed with follicular lymphoma (FL) in Finland 1995–2018. (A) Cumulative incidence of transformation for whole population (dash lines indicate the 95% confidence interval). (B) Cumulative incidence of transformation for different histological grades of FL.

Transformation occurred in 291 patients during 28 466 person‐years of follow‐up (crude transformation rate of 10.2/1000). The cumulative incidence of transformation for the entire cohort was 4.6% (95% confidence interval [CI], 3.9–5.3) at 5 years and 8.4% at 10 years (95% CI, 7.5–9.5) (Figure 1A). Of the 291 transformation events, 155 (53%) were in females. The median age at the time of transformation was 68 years (IQR, 61–75; range 21–94), with no significant difference between sexes.

At 5 years, the cumulative incidence of transformation was highest in grade 3A FL (6.4%; 95% CI, 4.0–10.2), followed by low‐grade FL (4.7%; 95% CI, 3.9–5.6) and unclassified FL (3.5%; 95% CI, 2.4–5.1) (Figure 1B), although the differences were not statistically significant.

HRs for incident transformation, adjusted for grade, sex, age and diagnosis year, are presented in Table 1. Older age at diagnosis significantly increased transformation risk (HR, 1.19 per 10‐year increase; 95% CI, 1.08–1.32; *p* < 0.001). No significant associations were seen for grade (HR, 1.27 for grade 3A vs. low‐grade; 95% CI, 0.81–1.99; *p* = 0.3), sex (HR, 0.82 for females vs. males; 95% CI, 0.65–1.03; *p* = 0.1) or diagnosis year (HR, 1.19 per 10‐year increase; 95% CI, 0.94–1.52; *p* = 0.2).

**TABLE 1:** Cox model for transformation in follicular lymphoma.

Characteristic	HR	95% CI	*p*–value
Age at diagnosis (per 10‐year increase)	1.19	1.08–1.32	<0.001
Sex			
Male	1.00	–	
Female	0.82	0.65–1.03	0.088
Grade			
Low‐grade	1.00	–	
Grade 3A	1.27	0.81–1.99	0.3
Unclassified	0.80	0.59–1.08	0.15
Year of diagnosis (per 10‐year increase)	1.19	0.94–1.52	0.15

Abbreviations: CI, confidence interval; HR, hazard ratio.

When examining survival after transformation, we found a significant interaction between calendar period and transformation status. Patients diagnosed in the later calendar period (2007–2018) had significantly higher mortality after transformation (HR, 2.09; 95% CI, 1.50–2.91; *p* < 0.001) compared to those diagnosed in the earlier period (1995–2006) (Table S2). The median time from FL diagnosis to transformation decreased from 7.7 years in the earlier period to 2.4 years in the later period.

To explore whether the risk of early transformation has changed over time, we repeated the analysis with follow‐up restricted to the first 5 years and stratified by diagnosis period. The 5‐year cumulative incidence remained approximately 3%–4% in each period (1995–2002, 2003–2008 and 2009–2013), and the adjusted cause‐specific HRs comparing later cohorts with earlier cohorts were not statistically significant (data not shown), indicating no secular trend in early transformation risk.

### Overall survival

During 29 361 person‐years of follow‐up, 1260 deaths occurred (crude mortality rate: 43/1000). OS was 80.3% (95% CI, 79.0%–81.7%) at 5 years and 65.0% (95% CI, 63.2%–66.8%) at 10 years.

Among 291 patients with transformation, 162 deaths occurred over 895 person‐years (crude mortality rate: 181/1000), while 1098 deaths occurred in patients without transformation over 28 466 person‐years (crude mortality rate: 39/1000). Median OS after transformation was 2.7 years.

After adjustment for age, sex, grade and the year of diagnosis (Table 2), transformation significantly increased total mortality (HR, 5.01; 95% CI, 4.21–5.96; *p* < 0.001). Mortality was also significantly higher in grade 3A FL patients compared to low‐grade (HR, 1.42; 95% CI, 1.13–1.78; *p* = 0.003). Furthermore, the risk of death was significantly lower with a more recent year of diagnosis (HR, 0.6 per 10‐year increase; 95% CI, 0.53–0.67; *p* < 0.001). The risk of death was comparable between females and males.

**TABLE 2:** Cox model for total mortality in follicular lymphoma.

Characteristic	HR	95% CI	*p*–value
Transformation	5.01	4.21–5.96	<0.001
Age at diagnosis (per 10‐year increase)	2.17	2.05–2.28	<0.001
Sex			
Male	1.00	–	
Female	0.72	0.64–0.80	<0.001
Grade			
Low‐grade	1.00	–	
Grade 3A	1.42	1.13–1.78	0.003
Unclassified	1.05	0.92–1.20	0.5
Year of diagnosis (per 10‐year increase)	0.60	0.53–0.67	<0.001

Abbreviations: CI, confidence interval; HR, hazard ratio.

**FIGURE 2:**
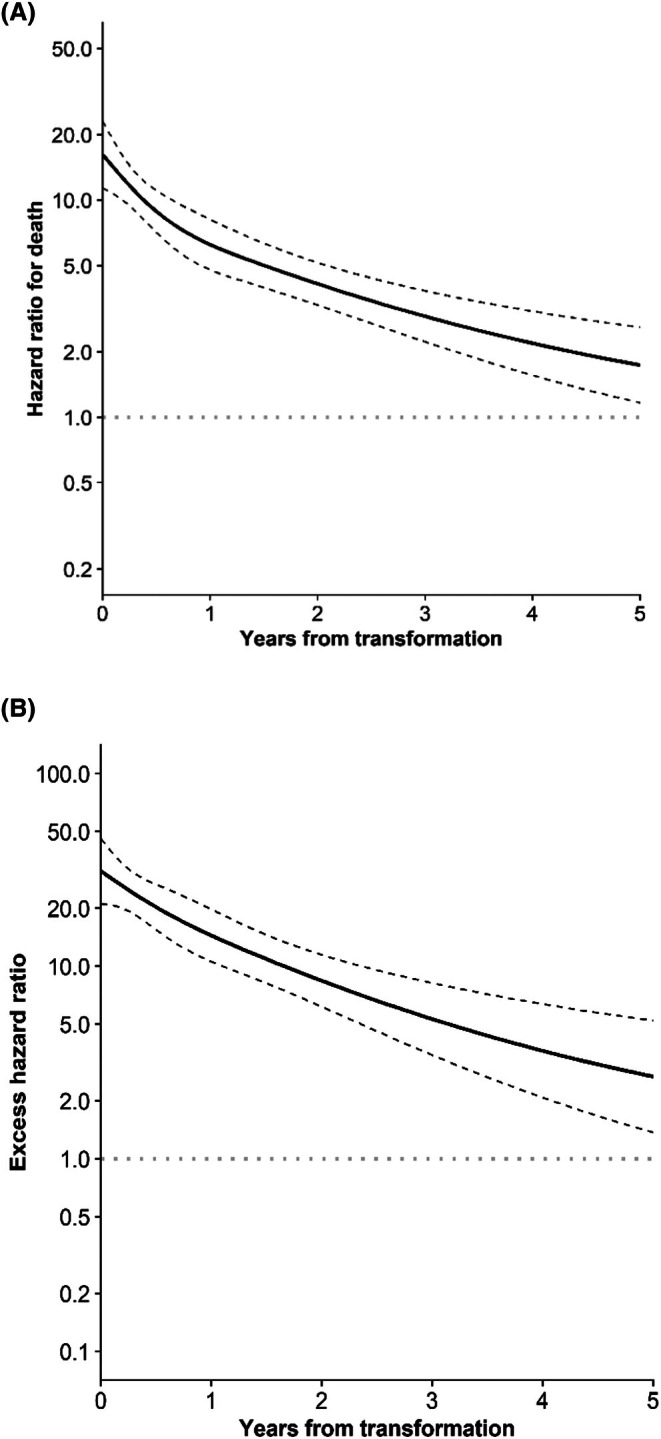
Hazard ratio for (A) mortality (on a logarithmic scale on the *y*‐axis) and for (B) excess mortality by the time from transformation. The dashed lines indicate 95% confidence interval.

The relative risk of death was highest shortly after transformation compared with patients without transformation (Figure 2A). The time‐dependent HRs at 1, 2 and 5 years following the transformation were 6.3 (95% CI, 4.8–8.2), 4.1 (95% CI, 3.3–5.2) and 1.7 (95% CI, 1.2–2.6) respectively.

### Relative survival

**FIGURE 3:**
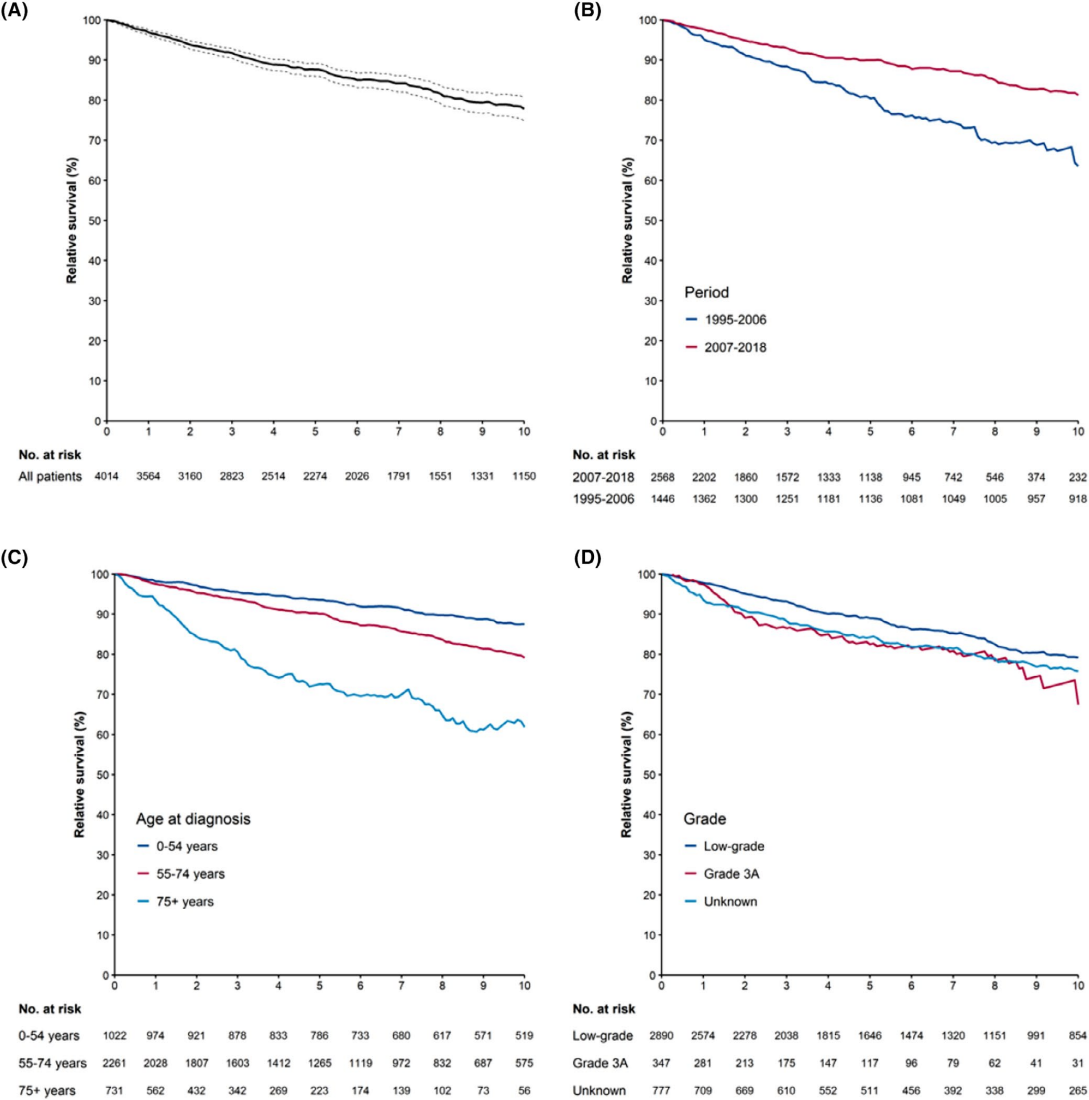
Relative survival (RS) in patients diagnosed with follicular lymphoma (FL) in Finland in 1995–2018. (A) Ten‐year age‐standardized RS during 1995–2018; (B) Ten‐year age‐standardized RS during 1995–2006 and 2007–2018; (C) Ten‐year age‐specific RS; (D) Ten‐year age‐standardized RS by FL grade (low‐grade: Grade 1–2).

The 10‐year age‐standardized RS of FL patients from 1995 to 2018 was 78% (95% CI, 75–81) (Figure 3A). By period, the 10‐year age‐standardized RS was 81% (95% CI, 78%–92%) for 2007–2018, compared to 64% (95% CI, 54%–72%) for 1995–2006 (Figure 3B). When follow‐up was truncated at 5 years, age‐standardized RS was 78.7% (74.6%–82.3%) in 1995–2002, 88.9% (85.6%–91.5%) in 2003–2008 and 90.1% (87.1%–92.4%) in 2009–2013 (Figure S1). RS was significantly lower in patients ≥75 years at diagnosis compared to younger patients (Figure 3C). Low‐grade FL patients had a higher 10‐year age‐standardized RS of 79% (95% CI, 76–82) vs. 67% (95% CI, 53–78) for grade 3A patients (Figure 3D).

Adjusted HRs for excess mortality are shown in Table 3. Older age at diagnosis and grade 3A increased excess mortality by 1.7‐fold (95% CI, 1.52–1.85; *p* < 0.001) and a 1.9‐fold (95% CI, 1.30–2.64; *p* < 0.001) respectively. Later diagnosis years reduced excess mortality risk (HR, 0.51 per 10‐year increase; 95% CI, 0.42–0.61; *p* < 0.001). No significant differences in excess mortality were observed between males and females. Transformation greatly increased excess mortality (HR, 13.1; 95% CI, 10.2–16.8; *p* < 0.001), although the excess relative risk declined over time (Figure 2B).

**TABLE 3:** Flexible parametric model for excess mortality in follicular lymphoma.

Characteristic	HR	95% CI	*p*–value
Transformation	13.1	10.2–16.8	<0.001
Age at diagnosis (per 10‐year increase)	1.67	1.52–1.85	<0.001
Sex			
Male	1.00	–	
Female	0.86	0.70–1.05	0.14
Grade			
Low‐grade	1.00	–	
Grade 3A	1.85	1.30–2.64	<0.001
Unclassified	1.16	0.92–1.46	0.2
Year of diagnosis (per 10‐year increase)	0.51	0.42–0.61	<0.001

Abbreviations: CI, confidence interval; HR, hazard ratio.

### Causes of death

Of the 1260 deaths recorded, 698 (55%) were attributed to lymphoma, 177 (14%) to secondary malignancies (SMNs) and 385 (31%) to other causes. Lymphoma was the leading cause of death in low‐grade (54%) and grade 3A FL (64%). SMNs accounted for 14% and 8%, and other causes for 31% and 27% respectively. In the subgroup of 291 patients who experienced transformation, lymphoma was the primary cause in 143 deaths (87%).

**FIGURE 4:**
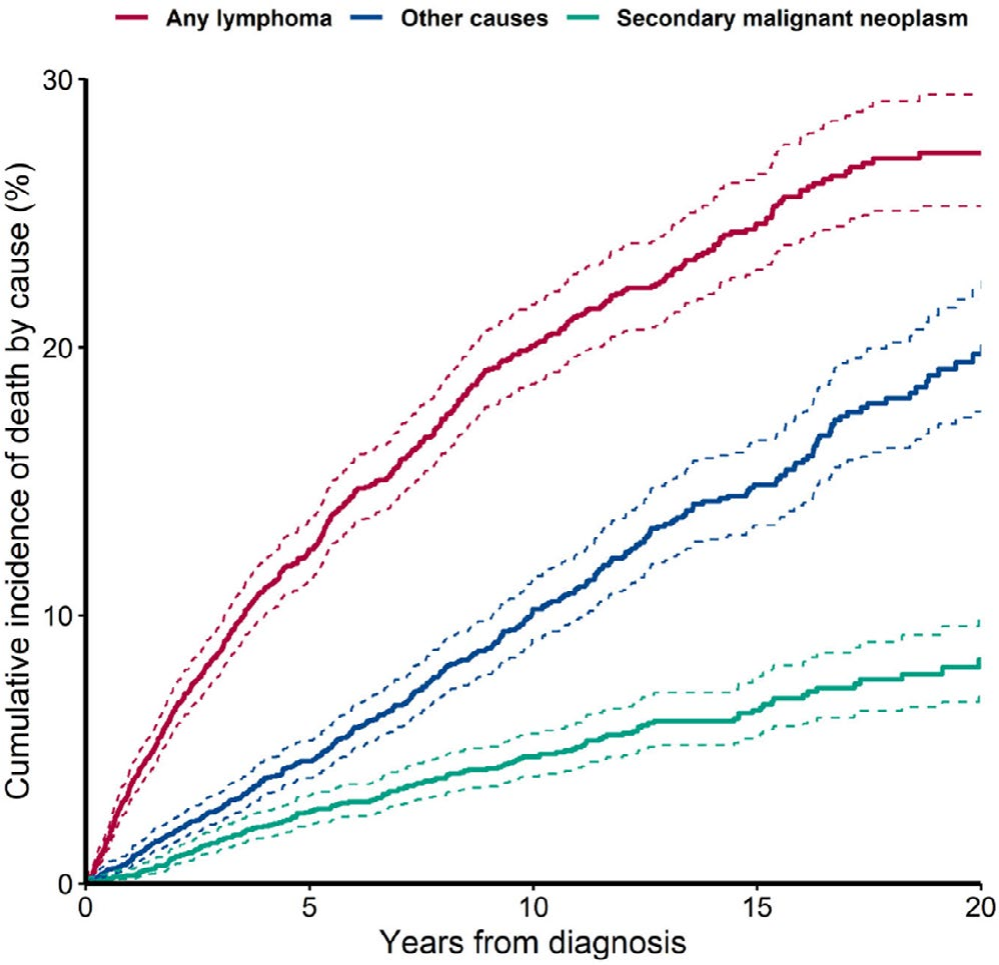
Cumulative incidence for the competing risks of cause of death.

Throughout follow‐up, lymphoma remained the leading cause of death, with a 20‐year cumulative incidence of 27.3% (95% CI, 25.3–29.4), followed by other causes at 19.7% (95% CI, 17.6–22.1). The 20‐year cumulative risk of SMN was 8.4% (95% CI, 7.0–10.0) (Figure 4).

## DISCUSSION

This large, nationwide study showed a 10‐year RS of FL at 78%. Prognosis improved in 2006–2018 compared to patients diagnosed in 1995–2005, even though patients diagnosed in the later period were significantly older and had a higher proportion of grade 3A FL. When follow‐up was restricted to 5 years, the age‐standardized 5‐year RS also increased across cohorts, rising from 78.7% in 1995–2002 to 90.1% in 2009–2013. The increase in grade 3A cases and the older age at diagnosis likely reflect improvements in pathological diagnostics, including more frequent diagnostic evaluations in older patients, as well as increased accuracy and completeness in case reporting to the FCR. The 10‐year cumulative transformation incidence was 8%. Transformation was associated with significant excess mortality. Excess mortality was also seen in older patients and in patients with grade 3A FL.

FL generally follows an indolent course with relatively good survival, as shown in our cohort and other large studies.^2^, ^13^, ^23^ The 10‐year OS in our study was 65%, which is lower than those reported by Sarkozy (77%–80%)^1^ and Batlevi (80%).^24^ However, patients in those studies were slightly younger (median ages of 59 and 57 years, respectively, compared to 64 years in our cohort), and neither study reported RS. In our cohort, the 10‐year RS was 78%, suggesting that a substantial proportion of long‐term deaths were unrelated to FL. In a Swedish cohort,^2^ younger patients had superior survival compared to older patients. Age at diagnosis, a well‐known adverse prognostic factor in FL, is included in the follicular lymphoma international prognostic index,^25^ a widely used prognostic tool.

Whether grade 3A results in poorer prognosis than low‐grade FL remains debated. Grade 3A FL has shown inferior outcomes with less aggressive treatment than grade 3B FL,^3^, ^4^ but some studies suggest it behaves as indolently as low‐grade FL patients.^6^, ^26^ In our study, grade 3A was associated with increased excess mortality compared to low‐grade FL. Importantly, we did not observe a significant difference in the risk of transformation across histological grades.

Historically, FL transformation rates reached up to 60% within 8 years.^27^ Many studies have reported lower annual rates of 2%–3%, and 5‐year rates varying between 11% and 20%.^12^, ^13^, ^28^ However, in the most recent studies, the 5‐year transformation rate has been lower (5%–8%)^23^, ^24^, ^29^ and comparable to our 5‐year transformation rate of 5%. Transformations continued over time without apparent plateauing. Older age at diagnosis significantly increased transformation risk. In our cohort, the transformation rate remained consistent among patients diagnosed in more recent calendar periods, suggesting that the introduction of rituximab in the initial treatment of FL has not diminished the transformation rate in this cohort. This is somewhat controversial given that the transformation rates in more recent studies are reported to be lower than in older studies.

Consistent with previous studies, transformation was associated with inferior survival and high excess mortality.^12^, ^13^ Sarkozy's study reported higher transformation rates in grade 3A FL compared to low‐grade disease; however, this was not observed in our study.^12^ Other factors contributing to a higher transformation rate include elevated lactate dehydrogenase (LDH) levels, low haemoglobin, B‐symptoms and altered performance status at diagnosis.^12^, ^13^ Evidence on whether primary treatment affects the transformation rate is unclear. Some studies have indicated that more aggressive treatment at diagnosis has reduced the transformation rate,^13^, ^28^, ^30^ while Conconi et al^31^ found that the transformation rate was lower in patients who were initially only observed.

The main limitation of our study is the lack of data on treatment and baseline characteristics beyond age and sex. Additionally, the absence of relapse data prevents us from evaluating the impact of disease progression at 24 months (POD24), an important prognostic marker for OS. Other limitations are similar to those of retrospective registry studies, as the data relied on registry records without central pathology review and verification of cause of death.

In Finland, current national guidelines recommend biopsy confirmation of transformation. However, we cannot exclude the possibility that some patients in this cohort were clinically diagnosed with transformed lymphoma without biopsy confirmation, which means these transformations may not have been captured in our analysis. Indeed, we observed that the median time from FL diagnosis to transformation significantly decreased from 7.7 years in patients diagnosed between 1995 and 2006 to 2.4 years in those diagnosed between 2007 and 2018. Patients diagnosed in the more recent period (2007–2018) experienced significantly higher mortality after transformation compared to those diagnosed earlier (1995–2006). We believe this difference may be due to ascertainment bias caused by more systematic biopsy verification in recent years, rather than a real difference in clinical outcomes. For comparison, in the PRIMA study, the biopsy rate for relapsed disease was 42%, with 20.6% histologically confirmed as transformed.^12^

In conclusion, this large nationwide, population‐based cohort study found that the 10‐year RS for patients diagnosed with FL in Finland between 1995 and 2018 was 78%. Excess mortality decreased among FL patients diagnosed in more recent years. The risk of transformation to aggressive B‐cell lymphoma was 8% at 10 years, and transformation was strongly associated with a significantly increased risk of death. Lymphoma remained the most common cause of death.

## AUTHOR CONTRIBUTIONS

IK, SJ and SL designed and conceived the study. IK, TR and LV collected the data. IK and TR analysed the data and wrote the manuscript. TT supervised the statistical analyses. NM, SJ and SL supervised the study. All authors interpreted the data, critically reviewed and revised the manuscript, and approved the final version.

## CONFLICT OF INTEREST STATEMENT

SL declares consultation fees from AbbVie, BMS, Genmab, Incyte, Novartis, Roche and Sobi; Honoraria from Gilead, Incyte, Roche and Sobi; and research grants from BMS, Hutchmed, Genmab, Novartis and Roche, all outside of submitted work. TR declares consultation fees from AbbVie, BMS, Gilead and Incyte, and travel and congress expenses from Gilead and Roche, all outside of submitted work.

## ETHICS STATEMENT

This study was conducted in accordance with the Declaration of Helsinki. The study was approved by the National Institute for Health and Welfare (Dnro THL/1441/5.05.00/2019), Statistics Finland (Dnro TK‐53‐1172‐19) and the Helsinki University Hospital Institutional Review Board.

## PATIENT CONSENT STATEMENT

Written informed consent was waived due to the retrospective nature of the study and the de‐identification of the patient information.

## Supporting information


Table S1.
Table S2.
Figure S1.

## Data Availability

The data that support the findings of this study are available from the corresponding author upon reasonable request.
