# Supplementary material for: Transformation and causes of death in follicular lymphoma: A Finnish nationwide population‐based study
Source: Br J Haematol. 2025 Sep 25;207(6):2417–25. doi: 10.1111/bjh.70181 (PMC12710176; doi:10.1111/bjh.70181)
Supplement: Supplementary file 1 — Table S1. Table S2. Figure S1. [file BJH-207-2417-s001.docx]

**Transformation and causes of death in follicular lymphoma:**

**A Finnish nationwide population-based study**

**Kalashnikov et al**

**Supplementary Material**

**Supplement Table 1.** **Baseline characteristics of patients diagnosed with follicular lymphoma in Finland years 1995**–**2006.**

| **Characteristic** | **Overall**  N=4,014*^1^* | **1995–2006**  N=1,446*^1^* | **2007–2018**  N=2,568*^1^* | ***p*–value*^2^*** |
| --- | --- | --- | --- | --- |
| Age at diagnosis (years) | 63.9 (54.7–72.2) | 59.2 (51.2–69.3) | 65.7 (58.0–73.2) | <0.001 |
| Sex |  |  |  | 0.4 |
| Male | 1,793 (45%) | 632 (44%) | 1,161 (45%) |  |
| Female | 2,221 (55%) | 814 (56%) | 1,407 (55%) |  |
| Grade (known vs unknown) |  |  |  | <0.001 |
| Low-grade | 2,890 (89%) | 1,010 (97%) | 1,880 (86%) |  |
| Grade 3A | 347 (11%) | 29 (2.8%) | 318 (14%) |  |
| Unknown | 777 | 407 | 370 |  |
| Transformation during follow-up | 291 (7.2%) | 146 (10%) | 145 (5.6%) | <0.001 |
| Patients’ status at last follow-up |  |  |  | <0.001 |
| Cencored | 2,754 (69%) | 701 (48%) | 2,053 (80%) |  |
| Dead | 1,260 (31%) | 745 (52%) | 515 (20%) |  |
| Follow-up time (years) | 6.1 (2.4–11.0) | 13.0 (5.9–16.5) | 4.3 (1.8–7.5) | <0.001 |
|  |  |  |  |  |

*^1^* Median (Q1–Q3); n (%)

*^2^* Wilcoxon rank sum test; Pearson’s Chi-squared test

**Supplement Table 2. Likelihood-ratio tests for interactions.**

| **Interaction test** | **X*^2^*** | **df** | ***p*–value** |
| --- | --- | --- | --- |
| Calendar period x Age | 0.307 | 1 | 0.579 |
| Calendar period x Sex | 0.210 | 1 | 0.646 |
| Calendar period x Grade | 4.555 | 2 | 0.103 |
| Calendar period x Transformation | 18.468 | 1 | <0.001 |


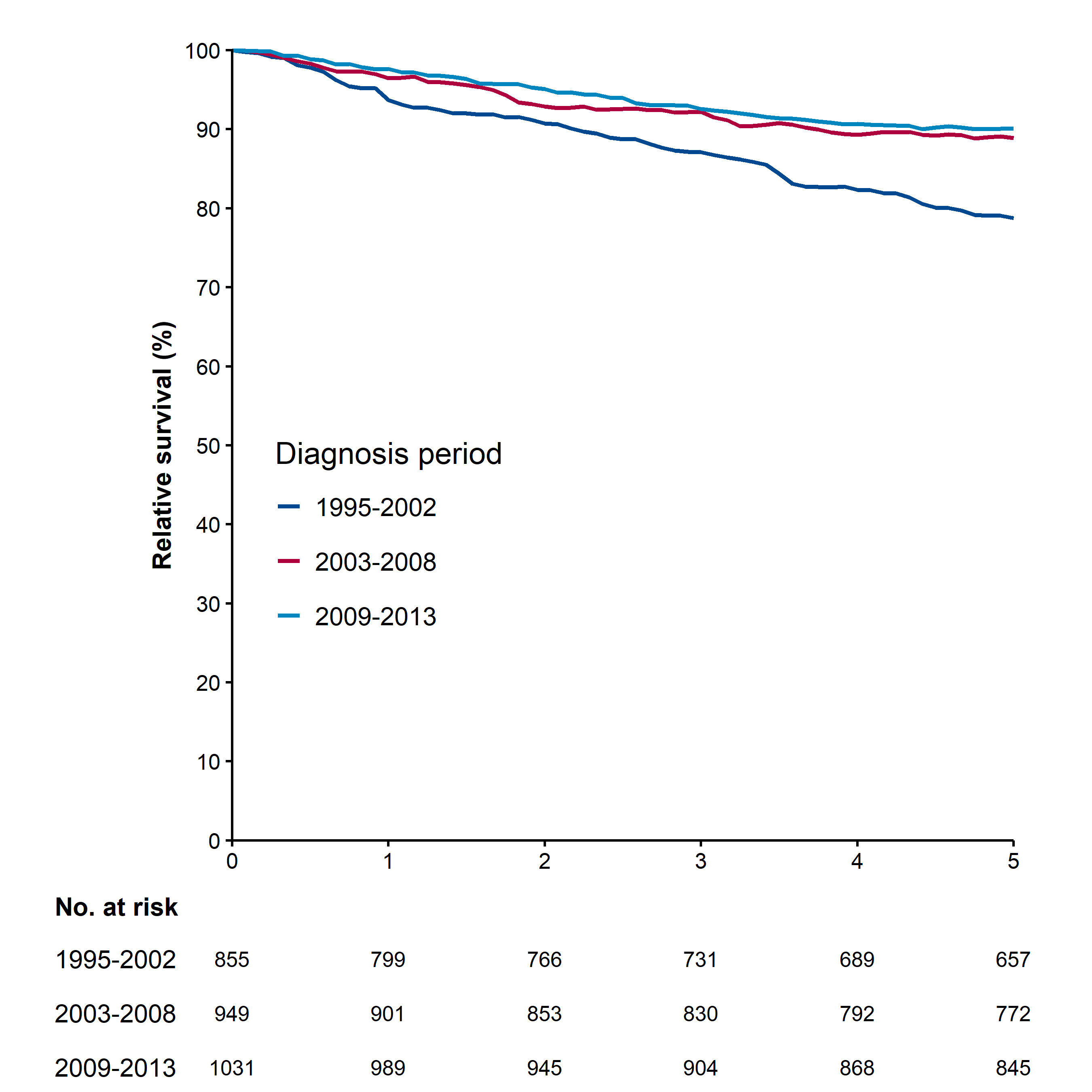


**Supplementary Figure 1. Age‑standardized five‑year relative survival (RS) in patients diagnosed with follicular lymphoma (FL) in Finland in 1995–2013. The figure shows RS curves by diagnosis period (1995–2002, 2003–2008, and 2009–2013) with follow‑up restricted to five years.**
